# Risk factors for disease progression and clinical outcomes in patients with COVID-19 in Taiwan: a nationwide population-based cohort study

**DOI:** 10.1186/s12890-024-03468-x

**Published:** 2025-01-26

**Authors:** Raymond N. Kuo, Wanchi Chen, Wen-Yi Shau

**Affiliations:** 1 Institute of Health Policy and Management, College of Public Health, National Taiwan University, 632R, No.17, Syujhou Rd., Taipei City 100, Taiwan; 2 Population Health Research Center, National Taiwan University, Taipei City, Taiwan; 3 Graduate Institute of Clinical Medicine, College of Medicine, National Taiwan University, Taipei City, Taiwan; 4 Graduate Institute of Clinical Pharmacy, College of Medicine, National Taiwan University, Taipei City, Taiwan

**Keywords:** COVID-19, Risk factors, Severity, Outcomes, Taiwan

## Abstract

**Background:**

Since 2021, COVID-19 has had a substantial impact on global health and continues to contribute to serious health outcomes. In Taiwan, most research has focused on hospitalized patients or mortality cases, leaving important gaps in understanding the broader effects of the disease and identifying individuals at high risk. This study aims to investigate the risk factors for disease progression through a nationwide population-based cohort study on COVID-19 in Taiwan.

**Methods:**

This study included 15,056 patients diagnosed with COVID-19 between January 1, 2021, and December 31, 2021, using the Taiwan National Health Insurance Research Database. Baseline and clinical characteristics were collected to verify the association with progression to severity outcomes, including hospital admission, intensive care unit (ICU) admission, invasive ventilatory support, fatal outcome, and the composite outcome of these four events. Patients were observed for 30 days for disease progression. Multivariable logistic regression models were used to calculate odd ratios and 95% confidence intervals (CIs) for each outcome, adjusting for age, sex, region, risk factors, and vaccination status.

**Results:**

Overall, 8,169 patients diagnosed during outpatient visits and 6,887 patients diagnosed during hospitalization were analyzed. Adjusting for age, sex, region, risk factors, and vaccination status, elderly patients had higher risks of hospital admission, ICU admission, invasive ventilatory support, fatal outcome, and composite outcome. Specifically, the risk of the fatal outcome was significantly higher for patients aged 75–84 (odds ratio: 6.11, 95% CI: 4.75–7.87) and those aged 85 years and older (12.70, 9.48–17.02). Patients with cardiovascular disease exhibited higher risks of hospital admission (1.60, 1.31–1.96), ICU admission (1.52, 1.31–1.78), invasive ventilatory support (1.57, 1.26–1.96), and fatal outcomes (1.26, 1.03–1.54) and the composite outcome (1.66, 1.20–1.54). Diabetes mellitus was identified as a significant risk factor for all clinical outcomes (hospital admission: 1.89, 1.53–2.35; ICU admission: 1.53, 1.30–1.79; invasive ventilatory support: 1.27, 1.01–1.60; the composite outcome: 1.45, 1.28–1.66), except for the fatal outcome.

**Conclusions:**

This study indicated the impact of sex, age, and risk factors on the clinical outcomes of COVID-19 patients in Taiwan. Elderly patients and those with cardiovascular disease or diabetes mellitus had higher risks for severe outcomes, including hospitalization, ICU admission, invasive ventilatory support, and mortality. These findings can provide evidence for a better understanding of risk factors for disease progression and inform targeted intervention.

## Background

COVID-19 has impacted individuals across diverse age spectrums, emerging as the primary contributor to hospitalizations and fatalities globally during the pandemic era. As of February 2024, the cumulative reported cases of COVID-19 worldwide have surpassed 774 million, with deaths exceeding 7 million [1]. In the initial phase of the pandemic in 2020, Taiwan managed to maintain a relatively low incidence of COVID-19 cases and transmission rates, attributed to the timely implementation of effective non-pharmaceutical interventions and an expedited public health response [2]. Nonetheless, this dynamic shifted following the advent of the alpha variant, culminating in a pronounced outbreak in May 2021. The subsequent identification of the delta variant in July 2021 and the emergence of the omicron variant in early 2022 further exacerbated the public health crisis. By the close of September 2023, Taiwan reported above 10 million confirmed cases and over 15,000 deaths [3].

The clinical spectrum of COVID-19 ranges from asymptomatic cases to severe illnesses characterized by respiratory failure, septic shock, and multi-organ dysfunction [4]. Approximately 20% of cases progress to a severe form, necessitating hospitalization, ventilation, and intensive care and often leading to significant morbidity and mortality [5]. Older age groups (aged over 65 years) and persons with underlying medical conditions, such as cardiovascular disease (CVD), chronic liver disease, chronic lung disease, chronic kidney disease, mental disorders, neurological diseases, overweight and tuberculosis (TB), are at-risk for severe clinical outcomes, including hospital admission, intensive care unit admission or death [6]. These risk factors related to severe clinical outcomes has been demonstrated in the national-wide studies from China, United States, and European countries [7–11]. A combined approach that includes prevention strategies with vaccines and anti-viral agents as complementary tools is supported by the World Health Organization [12]. The US National Institutes of Health (NIH) guidelines recommend a multifaceted approach to managing non-hospitalized patients with acute COVID-19, including supportive care, guidance for timely healthcare consultations, and COVID-19-specific therapy for high-risk individuals [13].

The development of therapies has seen the early emergence of vaccines, followed by promising drug candidates such as remdesivir, nirmatrelvir/ritonavir, and molnupiravir, which have demonstrated efficacy in improving clinical outcomes for COVID-19 patients at risk of severe manifestations due to underlying health conditions [14]. In Taiwan, vaccination campaigns were initiated on March 22, 2021, focusing primarily on priority groups such as older adults, long-term care residents, and healthcare workers, driven by the constraints imposed by limited vaccine supplies [15]. It wasn’t until April 21, 2022, that the first antiviral agent, specifically nirmatrelvir/ritonavir, became accessible for treating COVID-19 in Taiwan [16,17].

Throughout the COVID-19 pandemic, numerous studies have explored the various aspects of its impact in Taiwan. Several studies have highlighted Taiwan’s proactive measures in addressing the pandemic, encompassing swift policy responses and vaccination campaigns [15,18,19]. Additionally, research has been conducted on the epidemiological aspects of the COVID-19 outbreak in Taiwan and individuals’ mental health conditions and resilience strategies amid this unparalleled crisis [20,21]. The provision of universal healthcare in Taiwan, ensuring unrestricted access to healthcare services, including specialized and tertiary care, may play a pivotal role in mitigating the pandemic’s impact at the population level. While certain studies have utilized hospital record data to elucidate the progression of the disease among hospitalized individuals or have discussed deceased cases based on case reports issued by the Taiwan Centers for Disease Control (TCDC) [22–24], there is currently a lack of a comprehensive, nationwide analysis investigating the characteristics of COVID-19 patients and identifying those at increased risk for disease progression from a broad population perspective—encompassing both outpatient and inpatient care settings. As a result, the full scope of the potential burden of COVID-19 on the patient population in Taiwan remains unclear.

This study aims to examine the characteristics of COVID-19 patients and those who have experienced severe outcomes due to the infection, using the Taiwan National Health Insurance Research Database (NHIRD). It is anticipated that the findings derived from this study will offer significant insights conducive to the strategic allocation of resources and facilitate the preemptive recognition of individuals at high risk within the Taiwanese population.

## Methods

### Data sources

This observational cohort study focused on patients diagnosed with COVID-19 in Taiwan, utilizing data obtained from the Taiwan National Health Insurance Research Database (NHIRD). The NHIRD constitutes a nationwide claims database in Taiwan, containing patient-level claims data about 99.9% of the 23 million Taiwanese population [25,26]. Mortality data were gathered through linkage with the National Death Registry (NDR), and records of COVID-19 vaccination were obtained from the National Immunization Information System (NIIS) database. All data were thoroughly de-identified and the IDs of the patients were all encrypted using the same encryption algorithm to enable the cross-linking of data while ensuring privacy protection. This study received approval from the Joint Institutional Review Board of the Medical Research Ethical Foundation, Taipei, Taiwan (No. 23-S-011-1).

### Study population

The study included patients diagnosed with COVID-19 infection by Polymerase Chain Reaction (PCR) between January 1, 2021, and December 31, 2021, as recorded in the database. COVID-19 diagnosis was defined using either the ICD-10 code U07.1 (COVID-19, virus identified) or the reimbursement code NND009 (confirmed COVID-19). The index date was determined as the date of the initial COVID-19 diagnosis.

### Ascertainment of outcomes

The clinical outcomes of this study included hospital admission, intensive care unit (ICU) admission, invasive ventilatory support, mortality, and a composite outcome of these four events (i.e., patients experienced at least one of these four outcomes) within 30 days of the initial COVID-19 diagnosis. Data regarding deaths were obtained through linkage with the NDR. Other outcome variables were extracted from the NHIRD using specific reimbursement codes.

### Ascertainment of covariates

Covariates included age, sex, region, COVID-19 vaccination, and risk factors associated with severe COVID-19, as referred to in the Guidelines for Clinical Management of SARS-CoV-2 Infection [27]. These risk factors comprised clinical characteristics and underlying comorbidities, such as age ≥ 65 years, asthma, cancer, diabetes mellitus (DM), chronic kidney disease, cardiovascular disease (CVD) (excluding hypertension), chronic lung diseases (including interstitial lung disease, pulmonary embolism, pulmonary hypertension, bronchiectasis, and chronic obstructive pulmonary disease [COPD]), tuberculosis, chronic liver diseases (including cirrhosis, non-alcoholic fatty liver disease, alcoholic liver disease, and autoimmune hepatitis), disabilities (including attention-deficit/hyperactivity disorder [ADHD], cerebral palsy, cognitive defects, learning disabilities, and spinal cord injury), mental health conditions (including mood disorders and schizophrenia spectrum disorders), dementia, smoking (current and former), body mass index (BMI) ≥ 30 kg/m^2^ or > 95th percentile in adolescents aged 12–17 years, pregnancy and recent pregnancy (within 6 weeks after childbirth), immunodeficiency or immunosuppression (including human immunodeficiency virus [HIV], primary immunodeficiencies, solid organ or hematopoietic stem cell transplantation, and the use of corticosteroids or other immunosuppressive medications). All information regarding these covariates was retrieved from the Taiwan NHIRD. The comorbidities were identified using the ICD-10 codes. They were considered valid if the diagnostic codes were present at least twice in the records of outpatient clinics or at least once during hospitalization within 1 year prior to the index COVID-19 diagnosis. The vaccination status was retrieved from the NIIS database and summarized by the doses (i.e., 0, 1, ≥ 2) received during the year before the index date.

### Statistical analysis

Continuous variables were characterized by mean and standard deviation (SD). Categorical variables were described in terms of frequencies and percentages. For comparing numerical variables with categorical variables, we employed either the T-test or the Mann-Whitney test, depending on the data’s distribution. We used either the chi-squared test or Fisher’s exact test to compare categorical variables. The incidence proportion for each clinical outcome, except for hospital admission, was calculated as the number of new events per 1,000 patients among all patients diagnosed with COVID-19 in 2021. For hospital admission, the incidence proportion was estimated among patients diagnosed with COVID-19 in the outpatient setting in 2021. The incidence risk ratio for each clinical outcome was calculated based on the incidence proportion, with the incidence of patients having at least one risk factor as the numerator and the incidence of those without any risk factors as the denominator. Multivariable logistic regression analyses were utilized to investigate the relationship between demographic, clinical factors, vaccination status, and clinical outcomes. All models were adjusted for covariates, including gender, region, age group, risk factors associated with severe COVID-19, and vaccination status. The threshold for statistical significance was established at a p-value of less than 0.05. All statistical procedures were executed utilizing SAS software, version 9.4 (SAS Inc., Cary, NC, USA).

## Results

The study identified 15,056 patients with a diagnosis of COVID-19 recorded between January 1, 2021, and December 31, 2021, for inclusion in the analysis. Of these patients, 54.3% were diagnosed during an outpatient visit, while 45.7% were diagnosed during hospitalization. The distribution by gender indicated that 45.2% of the patients were male and 42.9% were female. A significant proportion of the diagnoses (80.4%) occurred in clinical settings within northern Taiwan. The average age at the initial diagnosis was 48.2 years, with a standard deviation of 20.4 years—roughly one-third of the cohort presented with at least one predefined risk factor for the progression of COVID-19. Among all the defined risk factors associated with severe COVID-19, the predominant risk factor was being aged 65 years or older (*n* = 3,678, accounting for 24.4% of cases), followed by the presence of CVD (excluding hypertension) (*n* = 1,359, 9.0%), DM (*n* = 1,241, 8.2%), chronic pulmonary disease (*n* = 551, 3.7%), and chronic kidney disease (*n* = 541, 3.6%). Most patients (86.9%) had not received the COVID-19 vaccine in 2021 (Table 1).

**Table 1:** Demographic characteristics and risk factors of all COVID-19 patients

	Total	
	(*N* = 15,056)	% (SD)
**Initial diagnosis of COVID-19 in clinical setting**		
Outpatient visit (including ER)	8,169	54.3
Hospitalization	6,887	45.7
**Gender**,** n (%)**		
Male	6,806	45.2
Female	6,456	42.9
Missing	1,794	11.9
**Region**,** n (%)**		
North	12,107	80.4
Middle	1,236	8.2
South	1,487	9.9
East	226	1.5
**Age at index date (year)**		
n	15,056	-
Mean (SD)	48.2	(20.4)
**Age group**,** n (%)**		
0–4	258	1.7
5–17	659	4.4
18–29	2,168	14.4
30–39	2,333	15.5
40–49	2,241	14.9
50–64	3,719	24.7
65–74	2,316	15.4
75–84	927	6.2
≥ 85	435	2.9
**Risk factors**,** n (%)**		
≥ 65 years old	3,678	24.4
Diabetes mellitus	1,241	8.2
Chronic kidney disease	541	3.6
Cardiovascular disease (excluding hypertension)	1,359	9.0
Chronic pulmonary disease	551	3.7
Immunodeficiency or immunosuppression	65	0.4
Malignancy	299	2.0
Tuberculosis	12	0.1
Chronic liver disease	136	0.9
Disabilities	51	0.3
Mental disease	202	1.3
Dementia	129	0.9
Asthma	196	1.3
Smoking (including current and former smokers)	8	0.1
Pregnancy and recent pregnancy (within 6 weeks after childbirth)	45	0.3
BMI ≥ 30 kg/m^2^ or > 95th percentile in adolescents aged 12–17 years	51	0.3
**Number of risk factors**,** n (%)**		
1	3,077	20.4
2	1,417	9.4
≥ 3	799	5.30
**Vaccination**,** n (%)**		
0	13,083	86.9
1	1,508	10.0
≥ 2	465	3.1

**Abbreviation**: COVID-19: Coronavirus Disease 2019; SD: Standard deviation

Within the subset of 8,169 individuals initially diagnosed with COVID-19 during an outpatient visit, 2,901 were subsequently hospitalized within 30 days, resulting in an incidence proportion of 355.1 per 1,000 individuals (95% Confidence Interval [CI]: 344.7-365.5). Across the entire study cohort, the incidence proportions per 1,000 were 92.9 (95% CI: 88.2–97.5) for admission to the ICU, 108.9 (95% CI: 100.1-117.6) for the requirement of invasive ventilatory support, 45.2 (95% CI: 41.9–48.6) for mortality and 287.8 (95% CI: 280.6–295.0) for the composite of these four outcomes. For patients exhibiting at least one risk factor for disease progression, the incidence risk ratios compared to patients without risk factors were 1.99 (95% CI: 1.89–2.11) for hospitalization post-outpatient diagnosis, 6.37 (95% CI: 5.64–7.19) for ICU admission, 2.48 (95% CI: 2.00-3.08) for the need of invasive ventilatory support, 14.27 (95% CI: 11.29–18.02) for mortality and 2.31 (95% CI: 2.20–2.42) for the composite outcome, as detailed in Table 2.

**Table 2:** Clinical outcomes of all COVID-19 patients

	Total (*N* = 15,056)	Patients with at least one risk factor	
		No	Yes
		(*N* = 9,763)	(*N* = 5,293)
**Hospital admission** ^**1, 2**^			
Yes	2,901	1,646	1,255
No	5,268	4,266	1,002
Incidence proportion, (95%CI)^3^	355.1 (344.7–365.5)	278.4 (267.0–289.8)	556.0 (535.6–576.5)
Incidence risk ratio, (95%CI)	--	Reference	1.99 (1.89–2.11)
**ICU admission** ^**2**^			
Yes	1,398	314	1,084
No	13,658	9,449	4,209
Incidence proportion, (95%CI)^3^	92.9 (88.2–97.5)	32.2 (28.7–35.7)	204.8 (193.9–215.7)
Incidence risk ratio, (95%CI)	--	Reference	6.37 (5.64–7.19)
**Invasive ventilatory support** ^**2**^			
Yes	525	94	431
No	14,531	9,669	4,862
Incidence proportion, (95%CI)^3^	108.9 (100.1–117.6)	55.4 (44.5–66.3)	137.8 (125.7–149.9)
Incidence risk ratio, (95%CI)	--	Reference	2.48 (2.00–3.08)
**Fatal outcome** ^**2**^			
Yes	681	78	603
No	14,375	9,685	4,690
Incidence proportion, (95%CI)^3^	45.2 (41.9–48.6)	8.0 (6.2–9.8)	113.9 (105.4–122.5)
Incidence risk ratio, (95%CI)	--	Reference	14.27 (11.29–18.02)
**Composite outcome** ^**2**^			
Yes	4,333	1,926	2,407
No	10,723	7,837	2,886
Incidence proportion, (95%CI)^3^	287.8 (280.6, 295.0)	197.3 (189.4, 205.2)	454.8 (441.3, 468.2)
Incidence risk ratio, (95%CI)	--	Reference	2.31 (2.20, 2.42)

^1^ Hospital admission was estimated among patients with COVID-19 diagnosed in the outpatient setting

^2^ Clinical outcomes were assessed within 30 days after initial diagnosis of COVID-19

^3^ New cases per 1,000 patients

**Abbreviation**: COVID-19: Coronavirus Disease 2019; CI: Confidence Interval; ICU: Intensive Care Unit

Table 3 illustrates the distribution of demographic characteristics, risk factors, and vaccination status of COVID-19 patients by clinical outcomes. The trends in hospital admissions varied based on patient characteristics. Within each gender and vaccination status subgroup, non-hospitalized patients were more prevalent. However, there is a noticeable trend where older age groups had a higher hospitalization proportion. Specifically, among patients aged 65–74, 75–84, and 85 years and older, more than half of the patients were hospitalized within 30 days of a COVID-19 diagnosis. Patients with certain comorbidities, such as tuberculosis (100%), dementia (86.7%), chronic pulmonary disease (67.8%), malignancy (67.7%), pregnancy (64%), DM (63.7%), smoking (62.5%), CKD (62.1%), cardiovascular disease (61.5%), mental disease (55.8%), chronic liver disease (53.1%) and immunodeficiency or immunosuppression (51.9%), exhibited higher hospitalization rates. Conversely, for ICU admission, invasive ventilatory support, and fatal outcomes, the proportion of patients without clinical outcomes was consistently higher across all demographics, comorbidities, risk factors, and vaccination statuses. For the composite endpoint, the older age groups were more prevalent, including those aged 75–84 (52.9%) and 85 years and older (58.6%). Regarding comorbidities, dementia (58.9%), chronic pulmonary disease (55.3%), CKD (54.7%), tuberculosis (50.5%), and smoking (50%) had a higher proportion of the composite outcome of these four events.

**Table 3:** Demographic characteristics, risk factors and vaccination status of COVID-19 patients by clinical outcomes

	Hospital admission^1^			ICU admission			Invasive ventilatory support			Fatal outcome			Composite outcome		
	Yes (*n* = 2,901)	No (*n* = 5,268)	*p*-value	Yes (*n* = 1,398)	No (*n* = 13,658)	*p*-value	Yes (*n* = 525)	No (*n* = 14,531)	*p*-value	Yes (*n* = 681)	No (*n* = 14,375)	*p*-value	Yes (*n* = 4,333)	No (*n* = 10,723)	*p*-value
	*n* (%)	*n* (%)		*n* (%)	*n* (%)		*n* (%)	*n* (%)		*n* (%)	*n* (%)		*n* (%)	*n* (%)	
**Gender**			< 0.001			< 0.001			< 0.001			< 0.001			< 0.001
Male	1,359 (37.6)	2,253 (62.4)		836 (12.3)	5,970 (87.7)		356 (5.2)	6,450 (94.8)		429 (6.3)	6,377 (93.7)		2,214 (32.5)	4,592 (67.5)	
Female	1,200 (32.9)	2,448 (67.1)		533 (8.3)	5,923 (91.7)		165 (2.6)	6,291 (97.4)		252 (3.9)	6,204 (96.1)		1,749 (27.1)	4,707 (72.9)	
Missing	342 (37.6)	567 (62.4)		29 (1.6)	1,765 (98.4)		4 (0.2)	1,790 (99.8)		0 (0.0)	1,794 (100.0)		370 (20.6)	1,424 (79.4)	
**Region**			< 0.001			< 0.001			< 0.001			< 0.001			< 0.001
North	1,940 (30.7)	4,384 (69.3)		1,193 (9.9)	10,914 (90.1)		489 (4.0)	11,618 (96.0)		638 (5.3)	11,469 (94.7)		3,242 (26.8)	8,865 (73.2)	
Middle	351 (49.9)	353 (50.1)		116 (9.4)	1,120 (90.6)		14 (1.1)	1,222 (98.9)		22 (1.8)	1,214 (98.2)		446 (36.1)	790 (63.9)	
South	514 (54.1)	436 (45.9)		79 (5.3)	1,408 (94.7)		20 (1.3)	1,467 (98.7)		17 (1.1)	1,470 (98.9)		544 (36.6)	943 (63.4)	
East	96 (50.3)	95 (49.7)		10 (4.4)	216 (95.6)		2 (0.9)	224 (99.1)		4 (1.8)	222 (98.2)		101 (44.7)	125 (55.3)	
**Age group**			< 0.001			< 0.001			< 0.001			< 0.001			< 0.001
0–4	25 (16.4)	127 (83.6)		6 (2.3)	252 (97.7)		0 (0.0)	258 (100.0)		≤ 3 (≤ 1.2)	≥ 255 (≥ 98.8)		31 (12.0)	227 (88.0)	
5–17	74 (17.0)	361 (83.0)		5 (0.8)	654 (99.2)		0 (0.0)	659 (100.0)		≤ 3 (≤ 0.5)	≥ 656 (≥ 99.5)		79 (12.0)	580 (88.0)	
18–29	402 (29.9)	944 (70.1)		26 (1.2)	2,142 (98.8)		4 (0.2)	2,164 (99.8)		≤ 3 (≤ 0.1)	≥ 2,165 (≥ 99.9)		426 (19.6)	1,742 (80.4)	
30–39	396 (26.8)	1,082 (73.2)		49 (2.1)	2,284 (97.9)		4 (0.2)	2,329 (99.8)		7 (0.3)	2,326 (99.7)		437 (18.7)	1,896 (81.3)	
40–49	375 (28.2)	954 (71.8)		96 (4.3)	2,145 (95.7)		27 (1.2)	2,214 (98.8)		9 (0.4)	2,232 (99.6)		455 (20.3)	1,786 (79.7)	
50–64	784 (40.0)	1,177 (60.0)		420 (11.3)	3,299 (88.7)		147 (4.0)	3,572 (96.0)		123 (3.3)	3,596 (96.7)		1,153 (31.0)	2,566 (69.0)	
65–74	526 (52.8)	471 (47.2)		482 (20.8)	1,834 (79.2)		218 (9.4)	2,098 (90.6)		219 (9.5)	2,097 (90.5)		1,007 (43.5)	1,309 (56.5)	
75–84	217 (66.8)	108 (33.2)		224 (24.2)	703 (75.8)		97 (10.5)	830 (89.5)		182 (19.6)	745 (80.4)		490 (52.9)	437 (47.1)	
85+	102 (69.9)	44 (30.1)		90 (20.7)	345 (79.3)		28 (6.4)	407 (93.6)		139 (32.0)	296 (68.0)		255 (58.6)	180 (41.4)	
**Risk factors**															
Diabetes mellitus	310 (63.7)	177 (36.3)	< 0.001	292 (23.5)	949 (76.5)	< 0.001	113 (9.1)	1,128 (90.9)	< 0.001	133 (10.7)	1,108 (89.3)	< 0.001	614 (49.5)	627 (50.5)	< 0.001
Chronic kidney disease	133 (62.1)	8 (37.9)	< 0.001	127 (23.5)	414 (76.5)	< 0.001	55 (10.2)	486 (89.8)	< 0.001	119 (22.0)	422 (78.0)	< 0.001	296 (54.7)	245 (45.3)	< 0.001
Cardiovascular disease (excluding hypertension)	338 (61.5)	212 (38.5)	< 0.001	322 (23.7)	1,037 (76.3)	< 0.001	135 (9.9)	1,224 (90.1)	< 0.001	183 (13.5)	1,176 (86.5)	< 0.001	668 (49.1)	691 (50.9)	< 0.001
Chronic pulmonary disease	137 (67.8)	65 (32.2)	< 0.001	178 (32.3)	373 (67.7)	< 0.001	48 (8.7)	503 (91.3)	< 0.001	46 (8.3)	505 (91.7)	< 0.001	305 (55.3)	246 (44.7)	< 0.001
Immunodeficiency or immunosuppression	14 (51.9)	13 (48.1)	0.076	7 (10.8)	58 (89.2)	0.680	≤ 3 (≤ 4.6)	≥ 62 (≥ 95.4)	0.496	≤ 3 (≤ 4.6)	≥ 62 (≥ 95.4)	0.231	21 (32.3)	44 (67.7)	0.529
Malignancy	88 (67.7)	42 (32.3)	< 0.001	55 (18.4)	244 (81.6)	< 0.001	19 (6.4)	280 (93.6)	0.006	41 (13.7)	258 (86.3)	< 0.001	147 (49.2)	152 (50.8)	< 0.001
Tuberculosis	5 (100.0)	0 (0.0)	0.006	≤ 3 (≤ 25.0)	≥ 9 (≥ 75.0)	0.382	≤ 3 (≤ 25.0)	≥ 9 (≥ 75.0)	0.653	≤ 3 (≤ 25.0)	≥ 9 (≥ 75.0)	0.525	6 (50.0)	6 (50.0)	0.1164
Chronic liver disease	34 (53.1)	30 (46.9)	0.003	24 (17.6)	112 (82.4)	0.001	5 (3.7)	131 (96.3)	0.813	11 (8.1)	125 (91.9)	0.045	57 (41.9)	79 (58.1)	0.001
Disabilities	12 (35.3)	22 (64.7)	0.979	5 (9.8)	46 (90.2)	0.898	≤ 3 (≤ 5.9)	≥ 48 (≥ 94.1)	0.426	≤ 3 (≤ 5.9)	≥ 48 (≥ 94.1)	0.270	18 (35.3)	33 (64.7)	0.303
Mental disease	53 (55.8)	42 (44.2)	< 0.001	35 (17.3)	167 (82.7)	< 0.001	8 (4.0)	194 (96.0)	0.712	12 (5.9)	190 (94.1)	0.329	85 (42.1)	117 (57.9)	< 0.001
Dementia	39 (86.7)	6 (13.3)	< 0.001	25 (19.4)	104 (80.6)	< 0.001	6 (4.7)	123 (95.3)	0.462	32 (24.8)	97 (75.2)	< 0.001	76 (58.9)	53 (41.1)	< 0.001
Asthma	45 (45.5)	54 (54.5)	0.038	30 (15.3)	166 (84.7)	0.004	4 (2.0)	192 (98.0)	0.330	8 (4.1)	188 (95.9)	0.765	76 (38.8)	120 (61.2)	0.002
Smoking (including current and former smokers)	5 (62.5)	3 (37.5)	0.186	≤ 3 (≤ 37.5)	≥ 5 (≥ 62.5)	0.754	≤ 3 (≤ 37.5)	≥ 5 (≥ 62.5)	0.753	≤ 3 (≤ 37.5)	≥ 5 (≥ 62.5)	0.691	4 (50.0)	4 (50.0)	0.239
Pregnancy and recent pregnancy (within 6 weeks after childbirth)	16 (64.0)	9 (36.0)	0.003	5 (11.1)	40 (88.9)	0.673	≤ 3 (≤ 6.7)	≥ 42 (93.3)	0.409	≤ 3 (≤ 6.7)	≥ 42 (≥ 93.3)	0.124	20 (44.4)	25 (55.6)	0.020
BMI > = 30 kg/m^2^ or > 95th percentile in adolescents aged 12–17 years	11 (45.8)	13 (54.2)	0.290	7 (13.7)	44 (86.3)	0.095	4 (7.8)	47 (92.2)	0.102	≤ 3 (≤ 5.9)	≥ 48 (≥ 94.1)	0.209	17 (33.3)	34 (66.7)	0.472
**Number of risk factors**,** n (%)**			< 0.001			< 0.001			< 0.001			< 0.001			< 0.001
1	700 (49.3)	719 (50.7)		527 (17.1)	2,550 (82.9)		212 (6.9)	2,865 (93.1)		250 (8.1)	2,827 (91.9)		1,231 (40.0)	1,846 (60.0)	
2	357 (63.8)	203 (36.3)		343 (24.2)	1,074 (75.8)		146 (10.3)	1,271 (89.7)		216 (15.2)	1,201 (84.8)		738 (52.1)	679 (47.9)	
≥ 3	80 (50.0)	80 (50.0)		214 (26.8)	585 (73.2)		73 (9.1)	726 (90.9)		137 (17.1)	662 (82.9)		438 (54.8)	361 (45.2)	
**Vaccination status**,** n (%)**			< 0.001			< 0.001			< 0.001			< 0.001			< 0.001
0	2,496 (37.6)	4,151 (62.4)		1,336 (10.2)	11,747 (89.8)		505 (3.9)	12,578 (96.1)		655 (5.0)	12,428 (95.0)		3,880 (29.7)	9,203 (70.3)	
1	284 (24.3)	885 (75.7)		56 (3.7)	1,452 (96.3)		18 (1.2)	1,490 (98.7)		21 (1.4)	1,487 (98.6)		329 (21.8)	1,179 (78.2)	
≥ 2	121 (34.3)	232 (65.7)		6 (1.3)	459 (98.7)		2 (0.6)	463 (99.4)		5 (1.1)	460 (98.9)		124 (26.7)	341 (73.3)	

^1^ Hospital admission was estimated among patients with COVID-19 diagnosed in the outpatient setting

The exact number of patients below 3 are not specified, in accordance with the regulations of NHIRD

**Abbreviation**: COVID-19: Coronavirus Disease 2019; ICU: Intensive Care Unit

The results of multivariable logistic regression analysis revealed significant associations between various risk factors and five clinical outcomes: hospital admission, ICU admission, invasive ventilatory support, fatal outcomes, and the composite outcome of these four events. Across all clinical outcomes, male patients exhibited a higher risk than females (adjusted OR for hospital admission: 1.29, 95%CI: 1.16–1.43; adjusted OR for ICU admission: 1.48, 95% CI: 1.30–1.67; adjusted OR for invasive ventilatory support: 2.00, 95% CI: 1.65–2.43; adjusted OR for fatal outcome: 1.67, 95% CI: 1.40–1.99; adjusted OR for the composite outcome: 1.27, 95% CI: 1.17–1.37).

Older cohorts, including patients aged 65–74, 75–84, and 85 years and older, were associated with higher risks than those aged 50 to 64. The trend of increasing risk with advancing age was observed across hospital admission, fatal outcomes, and the composite endpoint. Specifically, the risk in the fatal outcome was significant, with patients aged 75–84 experiencing a 6.11-fold increase (adjusted OR: 6.11, 95% CI: 4.75–7.87), and those aged 85 years and older experiencing a 12.70-fold increase (adjusted OR: 12.70, 95% CI: 9.48–17.02) in mortality (Table 4).

**Table 4:** Multivariable Analysis of Risk Factors Associated with Clinical outcomes: Hospital Admission, ICU admission, Invasive Ventilatory Support, Mortality, and Composite outcomes

	Hospital admission		ICU admission		Invasive ventilatory support		Mortality		Composite outcome	
	**OR (95% CI)** ^1^	**p-value**	**OR (95% CI)** ^1^	**p-value**	**OR (95% CI)** ^1^	**p-value**	**OR (95% CI)** ^1^	**p-value**	**OR (95% CI)** ^1^	**p-value**
**Gender**										
Male	1.29 (1.16–1.43)	< 0.001	1.48 (1.30–1.67)	< 0.001	2.00 (1.65–2.43)	< 0.001	1.67 (1.40–1.99)	< 0.001	1.27 (1.17–1.37)	< 0.001
Female	Reference		Reference		Reference		Reference		Reference	
**Region**										
North	Reference		Reference		Reference		Reference		Reference	
Middle	2.59 (2.19–3.06)	< 0.001	1.45 (1.17–1.81)	< 0.001	0.58 (0.37–0.92)	0.022	0.51 (0.32–0.80)	0.003	2.10 (1.83–2.39)	< 0.001
South	2.80 (2.41–3.24)	< 0.001	0.70 (0.55–0.90)	0.005	0.32 (0.19–0.56)	< 0.001	0.31 (0.19–0.52)	< 0.001	2.07 (1.84–2.34)	< 0.001
East	2.95 (2.17–4.01)	< 0.001	0.61 (0.31–1.19)	0.145	0.31 (0.07–1.25)	0.099	0.59 (0.21–1.66)	0.319	3.22 (2.43–4.26)	< 0.001
**Age group**										
0–4	0.34 (0.22–0.53)	< 0.001	0.19 (0.09–0.44)	< 0.001	N.A.		N.A.		0.33 (0.22–0.48)	< 0.001
5–17	0.36 (0.28–0.48)	< 0.001	0.07 (0.03–0.16)	< 0.001	N.A.		N.A.		0.34 (0.26–0.44)	< 0.001
18–29	0.65 (0.59–0.76)	< 0.001	0.11 (0.07–0.16)	< 0.001	0.06 (0.02–0.15)	< 0.001	0.03 (0.01–0.13)	< 0.001	0.55 (0.48–0.64)	< 0.001
30–39	0.55 (0.47–0.64)	< 0.001	0.19 (0.14–0.25)	< 0.001	0.05 (0.02–0.13)	< 0.001	0.10 (0.05–0.21)	< 0.001	0.53 (0.47–0.61)	< 0.001
40–49	0.62 (0.53–0.72)	< 0.001	0.39 (0.31–0.49)	< 0.001	0.32 (0.21–0.49)	< 0.001	0.12 (0.06–0.25)	< 0.001	0.60 (0.53–0.69)	< 0.001
50–64	Reference		Reference		Reference		Reference		Reference	
65–74	1.60 (1.36–1.88)	< 0.001	1.82 (1.57–2.11)	< 0.001	2.23 (1.79–2.79)	< 0.001	2.78 (2.20–3.50)	< 0.001	1.60 (1.44–1.79)	< 0.001
75–84	2.45 (1.88–3.20)	< 0.001	2.03 (1.68–2.46)	< 0.001	2.37 (1.79–3.13)	< 0.001	6.11 (4.75–7.87)	< 0.001	2.17 (1.86–2.53)	< 0.001
85+	2.83 (1.89–4.24)	< 0.001	1.67 (1.27–2.20)	< 0.001	1.46 (0.95–2.26)	0.086	12.70 (9.48–17.02)	< 0.001	2.71 (2.18–3.36)	< 0.001
**Risk factors** ^**2**^										
Diabetes mellitus	1.89 (1.53–2.35)	< 0.001	1.53 (1.30–1.79)	< 0.001	1.27 (1.01–1.60)	0.043	0.89 (0.71–1.11)	0.298	1.45 (1.28–1.66)	< 0.001
Chronic kidney disease	1.30 (0.94–1.79)	0.111	1.27 (1.01–1.59)	0.039	1.32 (0.97–1.81)	0.082	2.31 (1.80–2.97)	< 0.001	1.51 (1.25–1.82)	< 0.001
Cardiovascular disease (excluding hypertension)	1.60 (1.31–1.96)	< 0.001	1.52 (1.31–1.78)	< 0.001	1.57 (1.26–1.96)	< 0.001	1.26 (1.03–1.54)	0.025	1.36 (1.20–1.54)	< 0.001
Chronic pulmonary disease	2.21 (1.59–3.06)	< 0.001	2.56 (2.10–3.14)	< 0.001	1.22 (0.89–1.69)	0.222	0.66 (0.47–0.92)	0.015	1.91 (1.59–2.30)	< 0.001
Immunodeficiency or immunosuppression	2.10 (0.93–4.70)	0.073	1.22 (0.52–2.88)	0.646	1.75 (0.52–5.91)	0.371	0.80 (0.18–3.54)	0.767	1.20 (0.69–2.10)	0.532
Malignancy	1.67 (1.11–2.52)	0.014	0.98 (0.71–1.34)	0.887	0.92 (0.56–1.49)	0.721	1.43 (0.98–2.08)	0.063	1.21 (0.94–1.54)	0.135
Tuberculosis	N.A.		0.33 (0.04–2.70)	0.298	N.A.		0.59 (0.07–5.26)	0.632	1.21 (0.35–4.22)	0.765
Chronic liver disease	1.27 (0.73–2.20)	0.396	1.29 (0.80–2.07)	0.297	0.63 (0.25–1.57)	0.318	1.28 (0.64–2.54)	0.484	1.18 (0.82–1.70)	0.378
Disabilities	1.00 (0.46–2.18)	0.994	1.05 (0.39–2.83)	0.918	N.A.		0.58 (0.13–2.61)	0.474	1.35 (0.72–2.53)	0.352
Mental disease	2.20 (1.41–3.42)	< 0.001	1.64 (1.11–2.42)	0.014	0.91 (0.44–1.88)	0.789	0.96 (0.51–1.81)	0.893	1.49 (1.11–2.01)	0.009
Dementia	4.39 (1.76–10.91)	0.002	0.86 (0.54–1.37)	0.524	0.51 (0.22–1.18)	0.114	1.37 (0.88–2.13)	0.169	1.33 (0.91–1.94)	0.136
Asthma	1.03 (0.66–1.61)	0.900	1.11 (0.73–1.71)	0.621	0.36 (0.13–0.98)	0.047	0.51 (0.24, 1.09)	0.084	1.17 (0.85–1.59)	0.340
Smoking (including current and former smokers)	1.92 (0.29–12.71)	0.499	0.86 (0.08–8.79)	0.899	N.A.		N.A.		1.82 (0.41–8.08)	0.429
Pregnancy and recent pregnancy (within 6 weeks after childbirth)	4.99 (2.15–11.57)	< 0.001	7.96 (3.01–21.08)	< 0.001	N.A.		N.A.		3.73 (2.04, 6.82)	< 0.001
BMI > = 30 kg/m^2^ or > 95th percentile in adolescents aged 12–17 years	1.88 (0.82–4.32)	0.134	2.16 (0.93–5.02)	0.074	3.44 (1.16–10.17)	0.026	2.28 (0.65–8.07)	0.201	1.53 (0.84–2.80)	0.164
**Vaccination status**										
0	Reference		Reference		Reference		Reference		Reference	
1	0.41 (0.35–0.48)	< 0.001	0.32 (0.24–0.43)	< 0.001	0.30 (0.19–0.48)	< 0.001	0.20 (0.13–0.32)	< 0.001	0.60 (0.52–0.68)	< 0.001
≥ 2	0.83 (0.65–1.06)	0.132	0.12 (0.05–0.28)	< 0.001	0.13 (0.03–0.50)	0.004	0.15 (0.06–0.38)	< 0.001	0.96 (0.77–1.20)	0.714

^1^ These analyses were adjusted for gender, region, age group, risk factors, and vaccination status [2]. The comorbidities were treated as dichotomous variables (with vs. without) and were identified using the diagnosis codes within 1 year prior to the index COVID-19 diagnosis

**Abbreviation**: COVID-19: Coronavirus Disease 2019; OR: Odds Ratio; CI: Confidence Interval; ICU: Intensive Care Unit; N.A.: Not available

The impact of risk factors and COVID-19 vaccination status varied by clinical outcomes (Table 4). For hospital admission, patients with DM (adjusted OR: 1.89, 95% CI: 1.53–2.35), cardiovascular disease (adjusted OR: 1.60, 95% CI: 1.31–1.96), chronic pulmonary disease (adjusted OR: 2.21, 95% CI: 1.59–3.06), malignancy (adjusted OR: 1.67, 95% CI: 1.11–2.52), mental disease (OR: 2.20, 95% CI: 1.41–3.42), dementia (adjusted OR: 4.39, 95%CI: 1.76–10.91) and pregnancy (adjusted OR: 4.99, 95% CI: 2.15–11.57) had higher risks. Vaccinated patients with one dose had lower risks of hospital admission (adjusted OR: 0.41, 95% CI: 0.35–0.48), but no significant effect was observed for patients with two or more doses compared to unvaccinated individuals. For ICU admission, patients with DM (adjusted OR: 1.53, 95% CI: 1.30–1.79), CKD (adjusted OR: 1.27, 95% CI: 1.01–1.59), cardiovascular disease (adjusted OR: 1.52, 95% CI: 1.31–1.78), chronic pulmonary disease (adjusted OR: 2.56, 95% CI: 2.10–3.14), mental disease (adjusted OR: 1.64, 95% CI: 1.11–2.42), and pregnancy (adjusted OR: 7.96, 95% CI: 3.01–21.08) had higher risks. Vaccinated patients had lower risks of ICU admission, with adjusted ORs of 0.32 (95% CI: 0.24–0.43) for one dose and 0.12 (95% CI: 0.05–0.28) for two or more doses compared to unvaccinated individuals. For invasive ventilatory support, patients with DM (adjusted OR: 1.27, 95% CI: 1.01–1.60), cardiovascular disease (adjusted OR: 1.57, 95% CI: 1.26–1.96), and BMI ≥ 30 kg/m² (adjusted OR: 3.44, 95% CI: 1.16–10.17) had higher risks, while patients with asthma (adjusted OR: 0.36, 95% CI: 0.13–0.98) had lower risks to the outcome. Vaccinated patients had lower risks of requiring invasive ventilatory support, with adjusted ORs of 0.30 (95% CI: 0.19–0.48) for one dose and 0.13 (95% CI: 0.03–0.50) for two or more doses compared to unvaccinated individuals. In terms of fatal outcomes, patients with CKD (adjusted OR: 2.31, 95% CI: 1.80–2.97) and cardiovascular disease (adjusted OR: 1.26, 95% CI: 1.03–1.54) had higher risks of mortality, while patients with chronic pulmonary disease (adjusted OR: 0.66, 95% CI: 0.47–0.92) had lower risks. Vaccinated patients had lower risks of mortality, with adjusted ORs of 0.20 (95% CI: 0.13–0.32) for one dose and 0.15 (95% CI: 0.06–0.38) for two or more doses compared to unvaccinated individuals. For the composite outcome, similar risk factors to those for ICU admission were observed, including DM (adjusted OR: 1.45, 95% CI: 1.28–1.66), CKD (adjusted OR: 1.51, 95% CI: 1.25–2.82), cardiovascular disease (adjusted OR: 1.36, 95% CI: 1.20–1.54), chronic pulmonary disease (adjusted OR: 1.91, 95% CI: 1.59–2.30), mental disease (adjusted OR: 1.49, 95% CI: 1.11–2.01), and pregnancy (adjusted OR: 3.73, 95% CI: 2.04–6.82) had higher risks. Only patients who received one dose of vaccine had a lower risk of the composite outcome (adjusted OR: 0.60, 95% CI: 0.52–0.68), while no significant effect was observed for patients with two or more doses compared to unvaccinated patients.

## Discussion

To our knowledge, this study is the first nationwide comprehensive analysis in Taiwan that details the characteristics of COVID-19 patients diagnosed in 2021. Utilizing the well-established National Health Insurance Research Database (NHIRD) and National Immunization Information System (NIIS), this study examined demographic characteristics, vaccination, the prevalence of risk factors for severe COVID-19 cases, and the incidence proportions of severe clinical outcomes. The findings indicated that most cases in the 2021 cohort were diagnosed in northern Taiwan. Approximately one-third of these cases exhibited at least one risk factor, and 87% of the patients were unvaccinated. The vaccination program was made available in Taiwan on March 22, 2021 ^15^. However, the common adverse reaction to the vaccination and scarce knowledge of COVID-19 vaccination reduced the motivation to be vaccinated in the beginning, which caused a comparably low vaccination rate that corresponded to our findings [28]. On the other hand, we could also find in the multivariable regression results that vaccination status showed a protective effect on severe clinical outcomes, echoing the current guidelines for COVID-19. The primary risk factors identified were being over the age of 65, CVD, and DM. Regarding the setting of diagnosis, a larger number of patients were diagnosed in outpatient settings, such as emergency rooms (ER), compared to hospital settings. Based on Taiwan’s soft lockdown and community screening policies during the COVID-19 outbreak in 2021, the outpatient diagnosis may mainly be attributed to the ER, considering the decline of outpatient routine visits across different level hospitals during the outbreak [29,30].

Based on the multivariable logistic regression analysis results, male patients exhibited a higher risk than females across all five clinical outcomes. Gender is a common confounding variable, and gender differences in the clinical outcomes of COVID-19 also occurred at all ages, with an overall higher burden in human males [31,32]. The consistent and reproducibility results from different studies suggest that while socio-economic factors may be influencing some aspects of the pandemic, fundamental differences in the immune response between males and females are likely to be a driving factor behind the significant sex-bias observed in the COVID-19 pandemic [31]. These findings were also consistent with previous nationwide studies from other countries [9,11,33]. Regarding comorbidities, cardiovascular disease showed statistically significant across five clinical outcomes, which indicated cardiovascular disease might be the most important risk factor, followed by DM. DM presented statistical significance across four clinical outcomes but the fatal outcomes. The association between cardiovascular disease, DM, and worse COVID-19 outcomes has been demonstrated in several meta-analysis studies [34–36]. The potential impacts of DM that cause severe COVID-19 outcomes might be through the following routes, including dysregulation of inflammatory pathways, immune function, and/or lung function [34]. DM, as a cardiovascular risk factor, may lead to immune function dysregulation, which may, in turn, increase susceptibility and predispose these patients to severe clinical outcomes of COVID-19 [37].

A critical aspect of our investigation is emphasizing a broader spectrum of risk factors for severe COVID-19 clinical outcomes [38], rather than solely focusing on comorbidities. This observation is consistent with recent literature reviews that have identified age, gender, and specific comorbid conditions as correlating with increased severity of COVID-19 and mortality risk [39]. Smaller-scale studies within Taiwan have also highlighted the correlation between advancing age and expedited progression to mortality, thereby emphasizing the substantial burden posed by the aging population in Taiwan. The multivariable regression analysis highlighted that the presence of risk factors significantly escalates the risk of severe clinical outcomes. This reinforces the critical importance of risk factors in determining the prognosis and clinical outcomes of COVID-19, contributing valuable insights to existing literature on the subject [40]. Other studies within Taiwan have documented the proportion of clinical outcomes, such as 27.8% for ICU admissions and 6.6% mortality among patients with the alpha variant, yet lacked a clear definition of what constitutes a clinical outcome. In contrast, our study explicitly defines clinical outcomes as events occurring within 30 days of diagnosis. The differences in defining risk factors, comorbidities, and clinical outcomes pose challenges in directly comparing our findings with previous studies. Despite these differences, our findings are consistent with the guidelines provided by the Taiwan CDC, reflecting the actual Taiwan situation from a national perspective and providing a comprehensive profile of COVID-19 patients in the region.

In 2021, the distribution of COVID-19 cases in Taiwan was predominantly in the northern region, following several local outbreaks that escalated to a level 3 alert, and most patients had not yet been vaccinated against COVID-19. This observation is consistent with the situational realities experienced during that time. Moreover, a notable fraction of patients initially diagnosed in outpatient settings necessitated hospitalization within 30 days. This development occurred against a backdrop where effective treatments were not available, and the widespread distribution of vaccines had not been achieved. Amid substantial community transmission and pressures on healthcare resources, the allocation of these resources was heavily influenced by symptom severity, as guided by governmental directives. As a result, certain patients diagnosed in outpatient facilities subsequently necessitated inpatient care. This scenario underscores the heightened demand for hospital resources in Taiwan during this time frame. The limitations on available resources and the prevailing policies may have led to delays in managing the disease, potentially exacerbating the community burden associated with the pandemic.

This study marks the first instance of leveraging the comprehensive national claims database at a population level to investigate the characteristics of COVID-19 patients in Taiwan for 2021. By applying stringent definitions and scientific rigor, most of our findings accurately reflect the circumstances surrounding the 2021 epidemic and are consistent with published references, validating the NHIRD utility for future investigations. Our results provide a representative snapshot of the patient landscape across Taiwan in 2021, as the analysis is based on a substantial, well-established claims database with universal coverage. Identifying risk factors for worsened COVID-19 prognosis is important to identify high-risk patient groups and target intervention strategies for clinical management in the future to improve outcomes for people following COVID-19. Nevertheless, this study is subject to several limitations. Primarily, the reliance on claims data in the NHIRD means that certain clinical information, such as height, weight, and BMI, cannot be directly obtained, requiring the use of proxy indicators like diagnostic codes for obesity or tobacco use counseling. Additionally, the database does not include records of COVID-19 symptoms. Despite these limitations, the impact on the study’s data quality is expected to be minimal, as key risk factors were identified using accurate diagnostic and procedure codes. Another limitation is that confirmed COVID-19 cases were identified by ICD-10 codes and reimbursement codes. This means that patients infected with COVID-19 but without defined confirmation, such as those who self-tested using home-based kits or were unaware of their infection, were excluded from the analysis. However, given that the variant during that period had a high fatality rate, the population had low vaccination coverage, and public health interventions were highly implemented, we expect the effect on the study’s results to be limited.

In conclusion, this study identified the association between risk factors defined by the Taiwan CDC and the progression of COVID-19 disease. The results reveal that age, gender, and specific comorbidities were associated with increased risk of severe COVID-19 outcomes and death. Additionally, this study also provided the incidence of severe COVID-19 outcomes when effective treatment was not available, and widespread vaccine distribution had not yet been achieved, identifying medication and vaccine requirements and potential gaps in this period in Taiwan. As this study has laid the foundation for further studies into COVID-19, future studies might include further investigations into the strategic allocation of resources, targeting vulnerable populations with risk factors, assessment of the effectiveness of COVID-19 treatment, and the potential protective effect of vaccines.

## Data Availability

This study is based on data from the National Health Insurance Research Database provided by the National Health Insurance Administration (NHIA), Ministry of Health and Welfare, and managed by the Health and Welfare Data Science Center (HWDC). The authors do not own the datasets and cannot prevent access to them. Some restrictions apply to the availability of these data, which were used under license for the current study and are not publicly available.

## Declarations

### Data sharing statement

This study is based on data from the National Health Insurance Research Database provided by the National Health Insurance Administration (NHIA), Ministry of Health and Welfare, and managed by the Health and Welfare Data Science Center (HWDC). The authors do not own the datasets and cannot prevent access to them. Some restrictions apply to the availability of these data, which were used under license for the current study and are not publicly available.

## References

[1] World Health Organization. WHO Coronavirus (COVID-19) Dashboard. Accessed October 25. 2023. https://covid19.who.int/

[2] Cheng HY, Liu DP. Early prompt response to COVID-19 in Taiwan: Comprehensive surveillance, decisive border control, and information technology support. J Formos Med Association Published Online November. 2022;11. 10.1016/j.jfma.2022.11.002. 10.1016/j.jfma.2022.11.002 PMC9650562 36424239

[3] Taiwan epidemic report. COVID-19 Dashboard. Accessed October 25. 2023. https://covid-19.nchc.org.tw/

[4] World Health Organization. 2019-nCoV outbreak is an emergency of international concern. January 31, 2020. Accessed January 1, 2022. https://www.euro.who.int/en/healthtopics/health-emergencies/coronavirus-covid-19/news/news/2020/01/2019-ncovoutbreak-is-an-emergency-of-international-concern

[5] Cummings MJ, Baldwin MR, Abrams D, et al. Epidemiology, clinical course, and outcomes of critically ill adults with COVID-19 in New York City: a prospective cohort study. Lancet. 2020;395(10239):1763–70. 10.1016/S0140-6736(20)31189-2. 32442528 10.1016/S0140-6736(20)31189-2 PMC7237188

[6] Centers for Disease Control and Prevention. Underlying Medical Conditions Associated with Higher Risk for Severe COVID-19: Information for Healthcare Professionals. February 9, 2023. Accessed October 23. 2023. https://www.cdc.gov/coronavirus/2019-ncov/hcp/clinical-care/underlyingconditions.html 34009770

[7] Guan Wjie, Liang W hua, Zhao Y et al. Comorbidity and its impact on 1590 patients with COVID-19 in China: a nationwide analysis. *The European Respiratory Journal*. 2020;55(5):2000547. 10.1183/13993003.00547-2020 10.1183/13993003.00547-2020 PMC7098485 32217650

[8] Gao Ydong, Ding M, Dong X, et al. Risk factors for severe and critically ill COVID-19 patients: a review. Allergy. 2021;76(2):428–55. 10.1111/all.14657. 33185910 10.1111/all.14657

[9] Rosenthal N, Cao Z, Gundrum J, Sianis J, Safo S. Risk factors Associated with In-Hospital mortality in a US National Sample of patients with COVID-19. JAMA Netw Open. 2020;3(12):e2029058. 10.1001/jamanetworkopen.2020.29058. 33301018 10.1001/jamanetworkopen.2020.29058 PMC7729428

[10] Warren-Gash C, Davidson JA, Strongman H, et al. Severe COVID-19 outcomes by cardiovascular risk profile in England in 2020: a population-based cohort study. Lancet Reg Health – Europe. 2023;27. 10.1016/j.lanepe.2023.100604. 10.1016/j.lanepe.2023.100604 PMC9991014 36911072

[11] Bergman J, Ballin M, Nordström A, Nordström P. Risk factors for COVID-19 diagnosis, hospitalization, and subsequent all-cause mortality in Sweden: a nationwide study. Eur J Epidemiol. 2021;36(3):287. 10.1007/s10654-021-00732-w. 33704634 10.1007/s10654-021-00732-w PMC7946619

[12] World Health Organization. WHO SAGE Roadmap for prioritizing uses of COVID-19 vaccines. November 10. 2023. Accessed November 20, 2023. https://www.who.int/publications/i/item/WHO-2019-nCoV-Vaccines-SAGE-Prioritization-2023.1

[13] National Institutes of Health. Outpatient Management of Acute COVID-19. COVID-19 Treatment Guidelines Web site. December 16, 2021. Accessed January 1. 2022. https://www.covid19treatmentguidelines.nih.gov/outpatient-management/

[14] Kumari M, Lu RM, Li MC, et al. A critical overview of current progress for COVID-19: development of vaccines, antiviral drugs, and therapeutic antibodies. J Biomed Sci. 2022;29(1):68. 10.1186/s12929-022-00852-9. 36096815 10.1186/s12929-022-00852-9 PMC9465653

[15] Sheng WH, Hsieh SM, Chang SC. Achievements of COVID-19 vaccination programs: Taiwanese perspective. *J Formos Med Assoc*. Published online April 27, 2023:S0929-6646(23)00143-2. 10.1016/j.jfma.2023.04.017 10.1016/j.jfma.2023.04.017 PMC10133881 37142477

[16] Ministry of Health & Welfare. The Taiwan Food and Drug Administration of the approved the import of Paxlovid. January 15, 2022. Accessed December 10. 2022. https://www.mohw.gov.tw/cp-5264-65592-1.html

[17] National Health Insurance Administration. List of antiviral drugs for treatment of COVID-19. June 8. 2022. Accessed December 10, 2022. http://sc-dr.tw/shangchan/upload/files/%E5%81%A5%E4%BF%9D%E7%BD%B2%E8%A1%8C%E6%94%BF%E5%8D%94%E5%8A%A9%E7%96%BE%E7%AE%A1%E7%BD%B2%E8%BE%A6%E7%90%86COVID-19%E6%8A%97%E7%97%85%E6%AF%92%E8%97%A5%E5%93%81.pdf

[18] Lai CC, Lee PI, Hsueh PR. How Taiwan has responded to COVID-19 and how COVID-19 has affected Taiwan, 2020–2022. J Microbiol Immunol Infect. 2023;56(3):433–41. 10.1016/j.jmii.2023.04.001. 37061349 10.1016/j.jmii.2023.04.001 PMC10079311

[19] Chen CW, Wei JCC. Employing digital technologies for effective governance: Taiwan’s experience in COVID-19 prevention. Health Policy Technol. 2023;12(2):100755. 10.1016/j.hlpt.2023.100755. 37287501 10.1016/j.hlpt.2023.100755 PMC10149355

[20] Chiang WC, Chen SH. Time attitudes affecting psychological health during COVID-19 pandemic: a two-wave, six-month prospective study in Taiwan. *Curr Psychol*. Published Online April. 2023;26:1–13. 10.1007/s12144-023-04643-9. 10.1007/s12144-023-04643-9 PMC10131497 37359570

[21] Wu HH, Su CH, Chien LJ, Tseng SH, Chang SC. Healthcare-associated COVID-19 outbreaks: a nationwide population-based cohort study. J Hosp Infect. 2022;124:29–36. 10.1016/j.jhin.2022.02.023. 35283225 10.1016/j.jhin.2022.02.023 PMC8907114

[22] Huang ACC, Lin SM, Chiu TH, et al. Comparison of clinical characteristics and outcomes of hospitalized patients infected with the D614G strain or alpha variant of COVID-19 in Taiwan: a Multi-center Cohort Study. Int J Med Sci. 2022;19(13):1912–9. 10.7150/ijms.76725. 36438919 10.7150/ijms.76725 PMC9682515

[23] Lin SM, Huang ACC, Chiu TH, et al. Clinical and laboratory predictors for disease progression in patients with COVID-19: a multi-center cohort study. Biomed J. 2023;46(1):100–9. 10.1016/j.bj.2022.11.002. 36414180 10.1016/j.bj.2022.11.002 PMC9674567

[24] Tsai SC, Chang WW, Lee WS. Analysis of an outbreak of COVID-19(alpha-variant) with rapid progression to mortality in Taipei, Taiwan. J Infect. 2022;84(1):e33–4. 10.1016/j.jinf.2021.11.009. 34800579 10.1016/j.jinf.2021.11.009 PMC8596654

[25] Lin LY, Warren-Gash C, Smeeth L, Chen PC. Data resource profile: the national health insurance research database (NHIRD). *Epidemiology and health*. 2018;40. 10.4178/epih.e2018062 PMC6367203 30727703

[26] Cheng TM. Taiwan’s new national health insurance program: genesis and experience so far. Health Aff (Millwood). 2003;22(3):61–76. 10.1377/hlthaff.22.3.61. 12757273 10.1377/hlthaff.22.3.61

[27] Taiwan Centers for Disease Control (Taiwan CDC). Guidelines for Clinical Management of SARS-CoV-2 Infection. 2023. https://www.cdc.gov.tw/Category/Page/xCSwc5oznwcqunujPc-qmQ

[28] Lin YJ, Yen CF, Chang YP, Wang PW. Comparisons of motivation to receive COVID-19 vaccination and related factors between Frontline Physicians and nurses and the Public in Taiwan: applying the Extended Protection Motivation Theory. Vaccines. 2021;9(5):528. 10.3390/vaccines9050528. 34065222 10.3390/vaccines9050528 PMC8160641

[29] Chan TC, Chou CC, Chu YC, et al. Effectiveness of controlling COVID-19 epidemic by implementing soft lockdown policy and extensive community screening in Taiwan. Sci Rep. 2022;12(1):12053. 10.1038/s41598-022-16011-x. 35835796 10.1038/s41598-022-16011-x PMC9282154

[30] Yan YH, Ho SYC, Chien TW, Chou W. Assessing the impact of COVID-19 on outpatient and inpatient revenues: a comparative analysis of large and small hospitals in Taiwan. Medicine. 2023;102(45):e35787. 10.1097/MD.0000000000035787. 37960821 10.1097/MD.0000000000035787 PMC10637565

[31] Peckham H, de Gruijter NM, Raine C, et al. Male sex identified by global COVID-19 meta-analysis as a risk factor for death and ITU admission. Nat Commun. 2020;11(1):6317. 10.1038/s41467-020-19741-6. 33298944 10.1038/s41467-020-19741-6 PMC7726563

[32] Binns CW, Lee MK, Doan TTD, Lee A, Pham M, Zhao Y. COVID and gender: a narrative review of the Asia-Pacific Region. Int J Environ Res Public Health. 2023;20(1):245. 10.3390/ijerph20010245. 10.3390/ijerph20010245 PMC9819659 36612567

[33] Li X, Xu S, Yu M, et al. Risk factors for severity and mortality in adult COVID-19 inpatients in Wuhan. J Allergy Clin Immunol. 2020;146(1):110–8. 10.1016/j.jaci.2020.04.006. 32294485 10.1016/j.jaci.2020.04.006 PMC7152876

[34] Harrison SL, Buckley BJR, Rivera-Caravaca JM, Zhang J, Lip GYH. Cardiovascular risk factors, cardiovascular disease, and COVID-19: an umbrella review of systematic reviews. Eur Heart J - Qual Care Clin Outcomes. 2021;7(4):330–9. 10.1093/ehjqcco/qcab029. 34107535 10.1093/ehjqcco/qcab029 PMC8294691

[35] Bae S, Kim SR, Kim MN, Shim WJ, Park SM. Impact of cardiovascular disease and risk factors on fatal outcomes in patients with COVID-19 according to age: a systematic review and meta-analysis. Heart. 2021;107(5):373–80. 10.1136/heartjnl-2020-317901. 33334865 10.1136/heartjnl-2020-317901

[36] Matsushita K, Ding N, Kou M, et al. The relationship of COVID-19 Severity with Cardiovascular Disease and its traditional risk factors: a systematic review and Meta-analysis. Global Heart. 2020;15(1):64. 10.5334/gh.814. 33150129 10.5334/gh.814 PMC7546112

[37] Driggin E, Madhavan MV, Bikdeli B, et al. Cardiovascular considerations for patients, Health Care Workers, and Health systems during the COVID-19 pandemic. J Am Coll Cardiol. 2020;75(18):2352. 10.1016/j.jacc.2020.03.031. 32201335 10.1016/j.jacc.2020.03.031 PMC7198856

[38] Laure Wynants B, Van Calster, Gary S, Collins, et al. Prediction models for diagnosis and prognosis of covid-19: systematic review and critical appraisal. BMJ. 2020;369:m1328. 10.1136/bmj.m1328. 32265220 10.1136/bmj.m1328 PMC7222643

[39] Izcovich A, Ragusa MA, Tortosa F, et al. Prognostic factors for severity and mortality in patients infected with COVID-19: a systematic review. PLoS ONE. 2020;15(11):e0241955. 10.1371/journal.pone.0241955. 33201896 10.1371/journal.pone.0241955 PMC7671522

[40] Ahrenfeldt LJ, Nielsen CR, Möller S, Christensen K, Lindahl-Jacobsen R. Burden and prevalence of risk factors for severe COVID-19 in the ageing European population – a SHARE-based analysis. Z Gesundh Wiss. 2022;30(9):2081–90. 10.1007/s10389-021-01537-7. 33868899 10.1007/s10389-021-01537-7 PMC8036158

